# High expression of SRSF1 facilitates osteosarcoma progression and unveils its potential mechanisms

**DOI:** 10.1186/s12885-024-12346-y

**Published:** 2024-05-12

**Authors:** Shuqi Li, Xinyi Huang, Shuang Zheng, Wenhui Zhang, Fang Liu, Qinghua Cao

**Affiliations:** 1 Department of Pathology, The First Affiliated Hospital, Sun Yat-sen University, Guangzhou, 510080 China; 2 Department of Pathology, The Seventh Affiliated Hospital, Sun Yat-sen University, Shenzhen, 518107 China; 3 State Key Laboratory of Organ Failure Research, Guangdong Provincial Key Laboratory of Viral Hepatitis Research, Department of Infectious Diseases, Department of Liver Tumor Center, Nanfang Hospital, Southern Medical University, Guangzhou, 510515 China; 4 Department of Oncology, Nanfang Hospital, Southern Medical University, Guangzhou, 510515 China

**Keywords:** Osteosarcoma, SRSF1, Tumor progression, RNA-sequence, Alternative splicing

## Abstract

**Background:**

SRSF1, a member of Serine/Arginine-Rich Splicing Factors (SRSFs), has been observed to significantly influence cancer progression. However, the precise role of SRSF1 in osteosarcoma (OS) remains unclear. This study aims to investigate the functions of SRSF1 and its underlying mechanism in OS.

**Methods:**

SRSF1 expression level in OS was evaluated on the TCGA dataset, TAGET-OS database. qRT-PCR and Western blotting were employed to assess SRSF1 expression in human OS cell lines as well as the interfered ectopic expression states. The effect of SRSF1 on cell migration, invasion, proliferation, and apoptosis of OS cells were measured by transwell assay and flow cytometry. RNA sequence and bioinformatic analyses were conducted to elucidate the targeted genes, relevant biological pathways, and alternative splicing (AS) events regulated by SRSF1.

**Results:**

SRSF1 expression was consistently upregulated in both OS samples and OS cell lines. Diminishing SRSF1 resulted in reduced proliferation, migration, and invasion and increased apoptosis in OS cells while overexpressing SRSF1 led to enhanced growth, migration, invasion, and decreased apoptosis. Mechanistically, Gene Ontology (GO) analysis, Kyoto Encyclopedia of Genes and Genomes (KEGG) analysis, and Gene Set Enrichment Analysis (GSEA) revealed that the biological functions of SRSF1 were closely associated with the dysregulation of the protein targeting processes, location of the cytosolic ribosome, extracellular matrix (ECM), and proteinaceous extracellular matrix, along with the PI3K-AKT pathway, Wnt pathway, and HIPPO pathway. Transcriptome analysis identified AS events modulated by SRSF1, especially (Skipped Exon) SE events and (Mutually exclusive Exons) MXE events, revealing potential roles of targeted molecules in mRNA surveillance, RNA degradation, and RNA transport during OS development. qRT-PCR confirmed that SRSF1 knockdown resulted in the occurrence of alternative splicing of SRRM2, DMKN, and SCAT1 in OS.

**Conclusions:**

Our results highlight the oncogenic role of high SRSF1 expression in promoting OS progression, and further explore the potential mechanisms of action. The significant involvement of SRSF1 in OS development suggests its potential utility as a therapeutic target in OS.

**Supplementary Information:**

The online version contains supplementary material available at 10.1186/s12885-024-12346-y.

## Introduction

Osteosarcoma (OS) is a highly malignant tumor that primarily affects the pediatric and adolescent populations. Efforts have largely centered on understanding the mechanisms associated with its metastases [1, 2], elucidating the molecular processes governing its development, and identifying potential therapeutic targets to reduce relapse and mortality rates [3–5].

In addition to traditional molecular pathological mechanisms, recent oncological studies have placed increased emphasis on alternative splicing (AS) – a cellular process responsible for generating multiple messenger RNA/protein isoforms from a single transcript [6, 7]. The splicing factor, a pivotal player in AS progression that orchestrates a multitude of gene transcripts and incites abnormal biological function, has been attributed as a potential harbinger of tumorigenesis [8].

Noteworthy within this context is the Serine/Arginine-Rich Splicing Factors (SRSFs), a group of structurally related proteins defined by an RS domain rich in arginine and serine residues, integral to efficient alternative RNA splicing [9]. Of particular interest is SRSF1, an exemplary splicing factor known to bind specifically to exonic enhancers and generate splicing variants [10]. Numerous studies have investigated the role of SRSF1 in tumorigenesis, with a focus on its involvement in transcriptional regulation mediated by lncRNA or microRNA in breast cancer [11–13], liver cancer, and lung cancers [14–16]. However, the association between SRSF1 and OS has received limited attention, and the precise biological functions of SRSF1 in OS remain largely unexplored. Therefore, this study aims to investigate the role of SRSF1 in the pathogenesis, progression, and development of OS, with a particular focus on its mechanisms and gene-splicing capabilities.

In this study, we conducted in vitro experiments to examine the cancer-related functions of SRSF1 in OS. We analyzed RNA sequences obtained from downregulated SRSF1 in human U2OS osteosarcoma cell lines to explore the underlying mechanisms. Our results provide compelling evidence suggesting that SRSF1 may act as an oncogenic factor, promoting the growth and progression of OS. Consequently, we propose that SRSF1 has the potential to serve as a novel and promising therapeutic target for OS.

## Methods and materials

### Cell culture

The human osteosarcoma cell lines (143B, MG63, HOS, U2OS) and bone marrow mesenchymal stem cells (BMSC) were purchased from ATCC (Manassas, VA, USA). Osteosarcoma cell lines were grown in DMEM (Gibco, NY, USA), and bone marrow mesenchymal stem cells were grown in low glucose-DMEM (Gibco, NY, USA), supplemented with 10% fetal bovine serum (PAN, seratech, Germany) and 100 U/ml penicillin/streptomycin solution (Gibco, NY, USA) at 37℃ in a humidified 5% CO2 atmosphere.

### Gene over-express or knockdown

The lentiviral vector used to overexpress SRSF1 was purchased from GENECHEM (Shanghai, China). Stable cancer cells were established after screening with puromycin (Beyotime Biotechnology, China). The small interfering RNAs used to knock down SRSF1 were manufactured by Ribobio (Guangzhou, China). The siRNA sequences are as follows. siSRSF1#1: CGACGGCTATGATTACGAT; siSRSF1#2: GCAGTTCGAAAACTGGATA; siSRSF1#3: GTACGGAAAGAAGATATGA. The second and the third sequence is used to knockdown SRSF1 for RNA-seq experiment. All the transient transfection was finally performed in HOS cells with the help of Lipofectamine 2000 reagent (Invitrogen, Carlsbad, CA). Small interfering RNAs were introduced into cells at a final concentration of 50 nM. The cells were harvested 48 h after transfection.

### RNA extraction and quantitative real-time PCR

Total RNA was extracted using Steady Pure Universal RNA Extraction Kit (ACCURATE BIOLOGY, China) according to the manufacturer’s instructions. The concentration and quality of the total RNA were assessed with Nanodrop One Spectrophotometer (Thermo Fisher Scientific, USA). According to the mRNA expression, reverse transcription was performed using Prime Script RT master mix (TaKaRa, Japan). Quantitative real-time PCR analysis was performed in triplicate on 7500 Fast Real-Time PCR System (Applied Biosystems, USA) using SYBR Premix Ex Taq (TaKaRa, Japan) and the expression level of GAPDH was used as endogenous control. To detect the efficiency of transient transfection, β-actin was used as endogenous control. Primers for SRSF1 (forward, 5’-TCTACTGACAGCCCCTTGGT-3’, reverse, 5’-ACTTCCAACTATGATTAGCACCCA-3’), GAPDH (forward, 5’-GCACCGTCAAGGCTGAGAAC-3’, reverse, 5’-TGGTGAAGACGCCAGTGGA-5’) and β-actin (forward, 5’-TGGCACCCAGCACAATGAA-3’, reverse, 5’-CTAAGTCATAGTCCGCCTAGAAGCA-3’). Primers for SRRM2 NM_016333.4 (forward, 5’-TTAAGCCAGGAGCCAGTGAAC-3’, reverse, 5’-CTCGGGAGACTTAGGTGGTGAA-3’) and SRRM2 XM_054379978.1 (forward, 5’- CCGTTCAACTTCTGCTGACTCT-3’, reverse, 5’-CGTGTCTTCCGAATGGTCTGT-3’). Primers for DMKN NM_001035516.4 (forward, 5’-TCTGCTCTGCTCCTGCTCCT-3’, reverse, 5’-GTAGTTCTGATCGTCTCTGCCTGC-3’), and DMKN NM_001190347.2 (forward, 5’-AATCTGGGATTCAGGGGCAAG-3’, reverse, 5’-TAGAGGAGGGCTCGGGTG-3’). Primers for SCAT1 NR_110848.1 (forward, 5’-CCTGGAATAGAAGATGCCTTGG-3’, reverse, 5’-TGCCTAACTTCCTCCTCTAACAA-3’), and SCAT1 NR_110849.1 (forward, 5’-GACTTCTCGTGGGCGTGAGTTT-3’, reverse, 5’-GACCTCGAATGCAACGTCTTCAGAT-3’). Results were analyzed using the 2–ΔΔct calculation method.

### Western blotting

Cells were lysed by using RIPA lysis buffer (CWBIO, China). After adding phosphatase inhibitor (100×) and Protease Inhibitor Cocktail (100×), the cell lysate underwent ultrasonication. The protein extracts were separated using 12% SDS gel electrophoresis and were then transferred to PVDF Western Blotting Membranes (Roche Diagnostics Gmbh Mannheim, Germany). After blocking with 5% skimmed milk, the membranes were incubated with primary antibodies of SRSF1 (1:1000, Proteintech), β-actin (1:1000, Proteintech), and GAPDH (1:1000, Cell Signaling), and cultured using secondary antibodies. Protein blots were cut prior to hybridisation with antibodies during blotting. Finally, the experimental results were visualized by using SAGECREATION and analyzed by Image J.

### Transwell assay

A Transwell assay was carried out to detect the migration and invasion capacity of HOS and U2OS osteosarcoma cell lines. The assays were performed in 8 μm pore size Transwell chambers (Corning, USA). A total of 100 µl transfected HOS (1.8 × 10^5^/ml) or U2OS (1.8 × 10^5^/ml) cell suspensions without FBS were seeded in chambers, which invasion group was also supplemented with serum-free medium and had an insert coated with Matrigel (Corning, USA). The bottom chamber was filled with DMEM containing 20% FBS. 18 h later, cells were immobilized with Paraformaldehyde and stained with 0.1% crystal violet (Beyotime Biotechnology, China). The experimental results were visualized with a bright-field microscope (Leica DMI4000B, Germany) and analyzed by Image J. And cells invaded in five randomly chosen fields were counted.

### Flow cytometry analysis (FCA)

Flow cytometry was used to detect apoptosis and cell cycle which were performed following the manufacturer’s protocol. In brief, cells were washed three times with cold PBS and then resuspended in 500 µl of 1× Binding Buffer, then 5 µl of APC Annexin V and 5 µl of propidium iodide (PI) were added to stain for 15 min at room temperature in the dark. Cells were analyzed by flow cytometry (Cyto FLEX S, Beckman, Germany). For cell cycle analysis, the cell suspension was first fixed with 70% cold ethanol for 3 h and then resuspended in 500 µl of 1× Binding Buffer. After adding 5 µl of propidium iodide (PI) and 15 min room temperature incubation, cells were analyzed by flow cytometry (Cyto FLEX 2, Beckman, Germany). All results were analyzed with FlowJo software (Tree Star).

### RNA-Seq

The sequencing data were analyzed with the assistance of Gene Chem. (Shanghai, China). Differentially expressed genes were analyzed by using Deseq2 software with *P* < 0.05 and |log2(fold change) | >1. The details of RNA-seq were declared in supplementary files.

### Statistical analysis

To compare the statistical significance between groups, a two-tailed Student’s t-test, and a one-way ANOVA test were used. It was considered significant when *P* < 0.05 of each difference. All statistical data were displayed as means ± standard deviation (SD) and analyzed for statistical significance with GraphPad Prism 8 (GraphPad Software, USA).

## Results

### The mRNA level of SRSF1 is high in the osteosarcoma tissues based on TCGA database

**Fig. 1:**
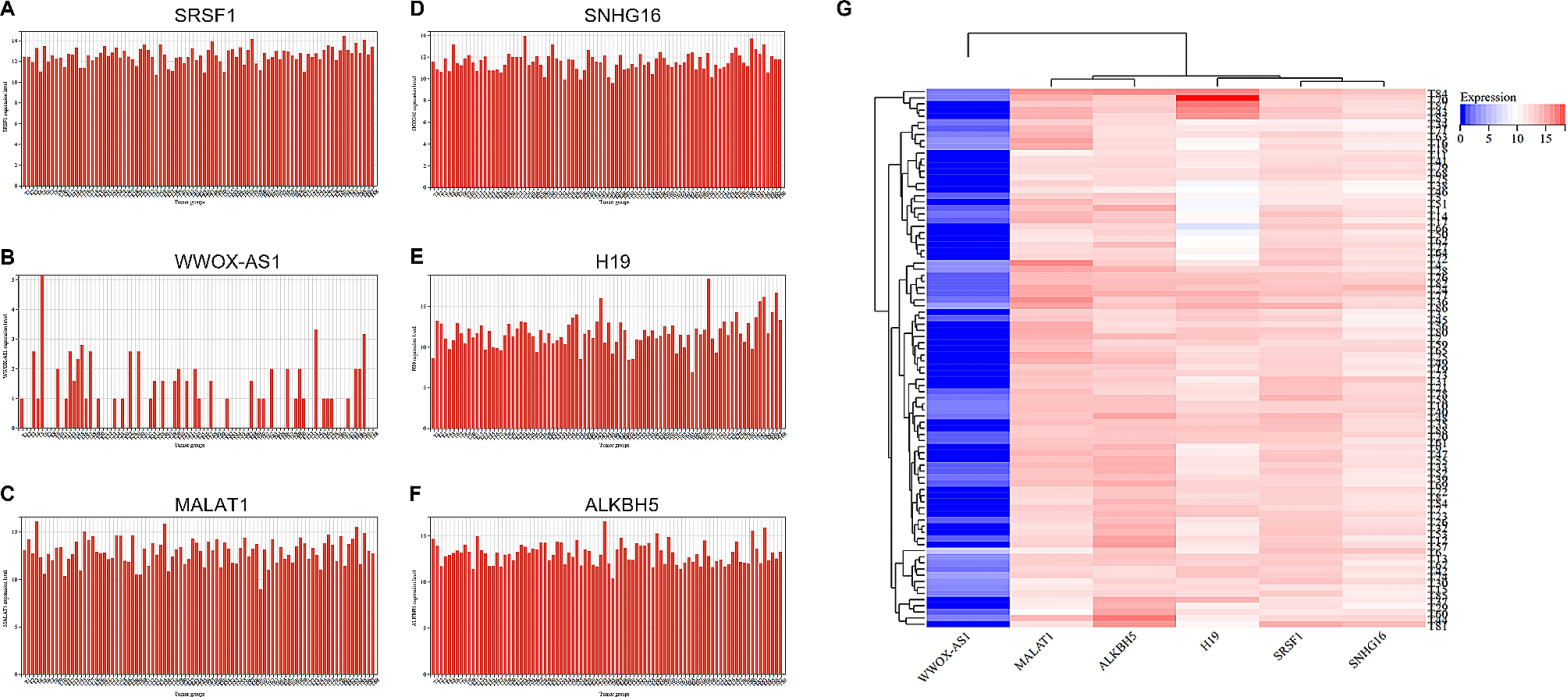
SRSF1 is highly expressed in the subset of the TCGA database. **(A)** SRSF1 is highly expressed in 88 tumor tissues based on TCGA database. **(B)** The expression of lncRNA WWOX-AS is remarkably low in 88 tumor tissues based on TCGA database. **(C)** MALAT1 is highly expressed in 88 tumor tissues based on TCGA database. **(D)** SNHG16 is highly expressed in 88 tumor tissues based on TCGA database. **(E)** H19 is highly expressed in 88 tumor tissues based on TCGA database. **(F)** ALKBH5 is highly expressed in 88 tumor tissues based on TCGA database. (**G**) Heatmap summarized six gene expressions and displayed the comparison of gene expression states according to the TAGET-OS database. These are showing normalized counts

Expression levels of SRSF1, as displayed by TAGET-OS, a subset of TCGA (http://xena.ucsc.edu/), were reported for 88 osteosarcoma tissues. Figure 1A indicates that SRSF1 is highly expressed in these 88 tumor tissues, with an average of 12.57. To corroborate our findings, we evaluated other known molecules in this database. We discovered that the expression of lncRNA WWOX-AS1 [17], which has confirmed antitumor effects, was remarkably low (with an average of 0.85) as depicted in Fig. 1B. Conversely, MALAT1 [18] (with an average of 12.95), lncRNA SNHG16 [19] (with an average of 11.56), lncRNA H19 [20](with an average of 11.76), and ALKBH5 [21] (with an average of 13.09), all known tumor promoters and have been confirmed higher expression in osteosarcoma samples and cells compared to normal samples and cells, expressed nearly as highly as SRSF1 in human osteosarcoma tissues (Fig. 1C-F). Altogether, the heatmap showed genes that were upregulated and downregulated in osteosarcoma tissues, implying a high expression state of SRSF1 and potential pathogenicity (Fig. 1G).

### SRSF1 promoted the proliferation, migration, and invasion and inhibited apoptosis of human osteosarcoma cells

**Fig. 2:**
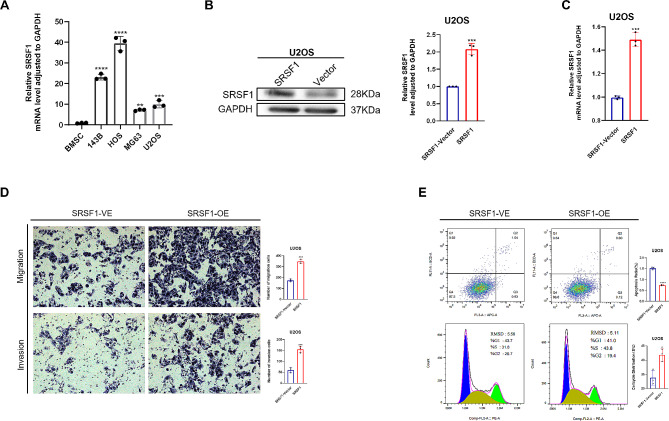
SRSF1 promoted the proliferation, migration, and invasion and inhibited apoptosis of human osteosarcoma cells (**A**) qRT-PCR detected SRSF1 mRNA levels in human osteosarcoma cell lines (143B, HOS, MG63, U2OS) and human bone marrow mesenchymal stem cells (BMSC). (**B**) Western blotting measured the efficiency of upregulating SRSF1 in protein-level U2OS cell lines. (**C**) qRT-PCR examined the effect of ectopic elevating SRSF1 in the mRNA level in U2OS cell lines. (**D**) Transwell assay investigated migration and invasion abilities while SRSF1 was overexpressed in U2OS cell lines. (**E**) Flow cytometry assessed the apoptosis ratio (top panels) and percentage of S phase in the cell cycle (bottom panels) that suggested active cell proliferation when increasing SRSF1 expression in U2OS cell lines. Each experiment was independently repeated three times. (*) *P* < 0.05; (**) *P* < 0.01; (***) *P* < 0.001; (****) *P* < 0.0001. Full-length blots/gels of SRSF1 and GAPDH in U2OS cell lines are presented in Figure S3 The grouping of blots cropped from different parts of the same gel

To determine the impact of SRSF1 on osteosarcoma development, we initially measured SRSF1 mRNA levels in four osteosarcoma cell lines and bone marrow mesenchymal stem cells. We employed quantitative real-time PCR methods and found that HOS had the most significantly elevated expression (a 39.4-fold increase), followed by 143B cells (a 23.3-fold increase), MG63 (a 7.2-fold increase) and U2OS (a 10-fold increase) registered increased levels compared to BMSC (Fig. 2A). Following this, we elevated SRSF1 expression in U2OS cell lines using lentivirus. Through the implementation of qRT-PCR and Western blotting techniques, we observed approximately a 1.5-fold elevation in SRSF1 levels, relative to the negative control, at the mRNA level, and a twofold augmentation in SRSF1 levels at the protein level (Fig. 2B-C). Subsequently, we first evaluated numerous phenotypes after elevating SRSF1 levels in U2OS human osteosarcoma cell lines. The transwell assay showed enhanced migration (a 2.1-fold increase) and invasion (a 2.8-fold increase) in comparison to negative controls (Fig. 2D). The flow cytometric analysis of cell apoptosis and cell cycle showed a reduction in cell apoptosis (∽ 50% reduction in SRSF1-OE) (Fig. 2E **upper panels**) and an increased S phase (1.2-fold) relative to negative controls during the cell cycle distribution when up-regulated SRSF1 gene expression (**Fig. 2E lower panels**). As a whole, our findings indicate that increasing SRSF1 boosts the proliferation, migration, and invasive capabilities of osteosarcoma cells and decreases cell apoptosis in vitro.

### Depletion of SRSF1 inhibited osteosarcoma cell proliferation, migration, and invasion, and promoted apoptosis in vitro

**Fig. 3:**
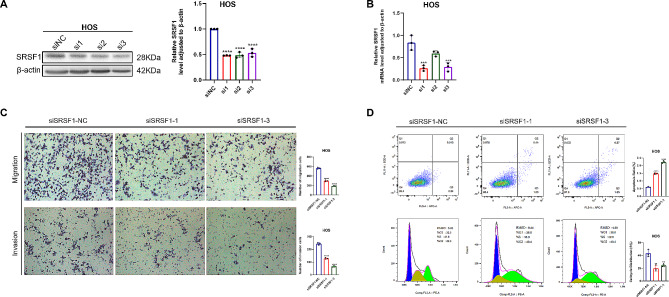
Depletion of SRSF1 inhibited osteosarcoma cell proliferation, migration, and invasion, and promoted apoptosis in vitro. (**A**) Western blotting measured the efficiency of downregulating SRSF1 in protein level in HOS. (**B**) qRT-PCR examined the effect of knockdown SRSF1 in the mRNA level in HOS. (**C**) Transwell assay investigated abilities of migration and invasion while SRSF1 was knockdown by two small interfering RNA in HOS cell lines. (**D**) Flow cytometry assessed the apoptotic cells (top panels) and each cell cycle phase (bottom panels) especially the S phase when downregulating SRSF1 in HOS cell lines. Each experiment was independently repeated three times. (*) *P* < 0.05; (**) *P* < 0.01; (***) *P* < 0.001; (****) *P* < 0.0001. Full-length blots/gels of SRSF1 and β-actin in HOS cell lines are presented in Figure S4.The grouping of blots cropped from different parts of the same gel

We then reduced SRSF1 expression in HOS cell lines using siRNA, accompanied by positive and negative controls. After qRT-PCR and Western blotting analyses, we selected two of the three most effective RNAi segments displayed in Fig. 3A and B, and Figure S1where they achieved reductions of ∽ 75% and ∽ 70% for siRNA-1 and siRNA-3, respectively, at the mRNA level and ∽ 53% and ∽ 48% at the protein level, respectively. Then we also assessed numerous phenotypes after diminishing SRSF1 levels in HOS cell lines. The transwell assay ascertained the abilities of migration and invasion. SRSF1 knockdown led to a decrease in the number of migrating (∽ 47% reduction in siRNA-1 and ∽ 67% reduction in siRNA-3) and invading cells (∽ 49% reduction in siRNA-1 and ∽ 74% reduction in siRNA-3) in comparison to negative controls (Fig. 3C). Then flow cytometric analysis of cell apoptosis and cell cycle showed downregulating SRSF1 levels caused a dramatic increase in apoptosis (2.6-fold in siRNA-1 and 3.9-fold in siRNA-3) relative to negative controls (Fig. 3D **upper panels**), and cell cycle analyses demonstrated decreased proportions of the S phase (∽ 57% reduction in siRNA-1 and ∽ 43% reduction in siRNA-3) in HOS cell lines (Fig. 3D **lower panels**). In all, our findings indicate that silencing SRSF1 appeared to hinder the proliferation, migration, and invasive capabilities of osteosarcoma cells and increase cell apoptosis in vitro.

### Summary of SRSF1-regulated gene expression in human U2OS osteosarcoma cell lines

**Fig. 4:**
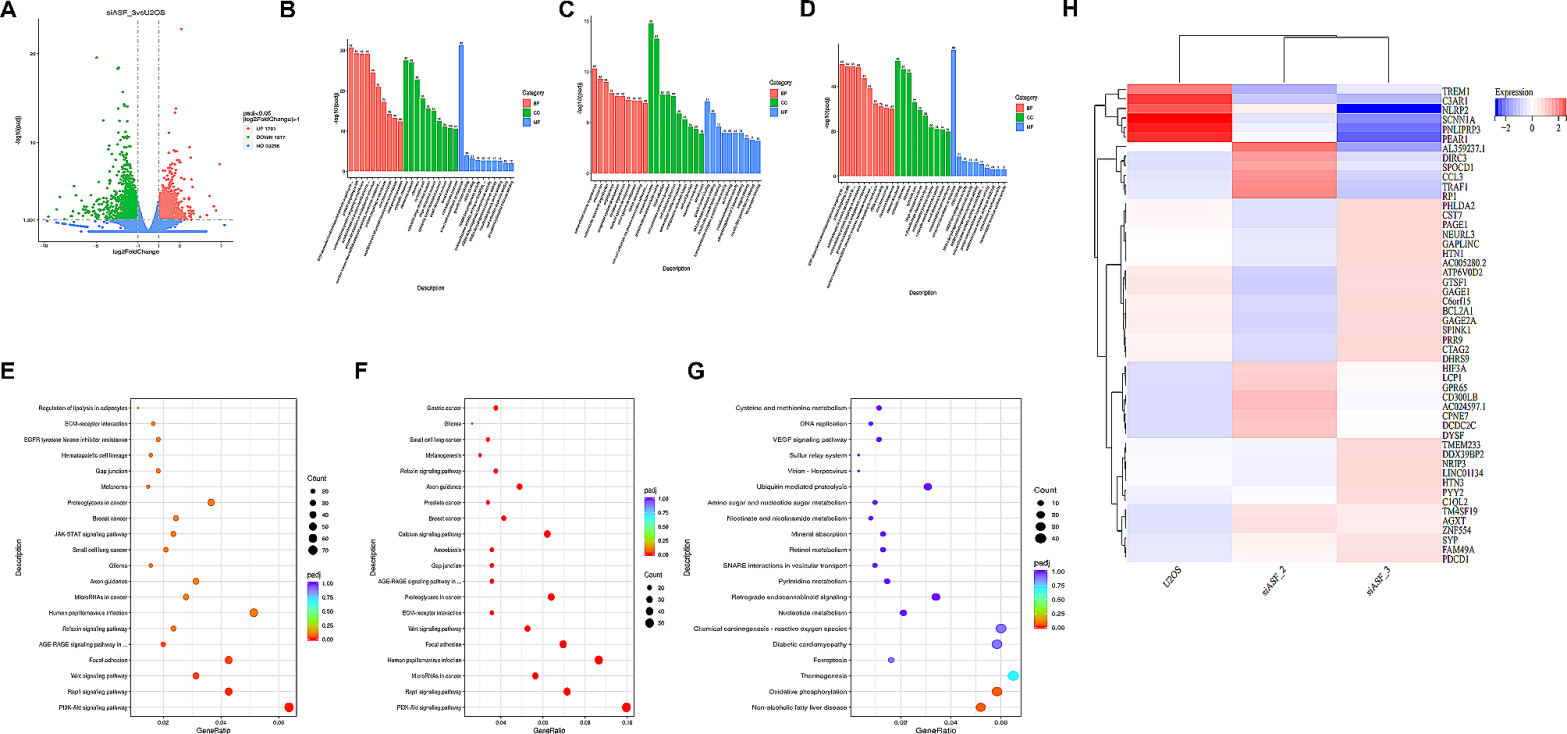
Summary of SRSF1-regulated gene expression in human U2OS osteosarcoma cell lines. (**A**) The volcano plot showed the up-regulated and down-regulated genes when SRSF1 was knockdown in U2OS compared with U2OS blank cells. (**B**) GO analyses investigated all genes’ biological functions in BP, CC, and MF layers in U2OS cell lines when SRSF1 knockdown. (**C**) Down-regulated genes’ biological data were clarified in BP, CC, and MF layers to display their biological functions in U2OS cell lines when SRSF1 knockdown. **(D**) GO analyses exhibited a biological functional field of up-regulated genes in BP, CC, and MF layers in U2OS cell lines with SRSF1 downregulated. (**E**) Bubble charts displayed the whole biological pathway changes in KEGG analyses in SRSF1-knockdown U2OS cells. (**F**) Metabolic pathways of down-regulated genes in SRSF1-knockdown U2OS cells were illustrated by KEGG analysis. (**G**) KEGG analysis provided information on the up-regulated genes’ expression within biological pathways in SRSF1-knockdown U2OS cells. (**H**) Clustered heat maps for 51 events regulated by SRSF1-targeted genes when knockdown SRSF1 in two sections in U2OS compared with U2OS blank cells in three experimental repeats

To decipher the cancer-promoting mechanisms of SRSF1, we examined changes in gene expression in human U2OS cells under conditions with and without SRSF1 knockdown. We conducted RNA-Seq, which revealed that 1701 genes were upregulated and 1317 genes were downregulated upon SRSF1 knockdown (Fig. 4A). TOP 100 upregulated and downregulated genes were respectively displayed in Supplementary Table S1-S2. The GO bar charts indicate the most discernible change in the protein targeting processes, the location of the cytosolic ribosome, and the function of a structural constituent of the ribosome (Fig. 4B). Regions such as the extracellular matrix (ECM) and the proteinaceous extracellular matrix appear to exhibit diminished presence (Fig. 4C). The structural components of the ribosomes have demonstrated enhanced functionality (Fig. 4D). Correspondingly, the KEGG bubble charts suggest that SRSF1 may influence multiple signaling pathways including PI3K − Akt, Rap1, Wnt, and focal adhesion (Fig. 4E). Notably, the activating of pathways signaling along with microRNA gene expression in cancers tend to decrease (Fig. 4F). Additionally, potential involvement in certain metabolic conditions such as non − alcoholic fatty liver disease and oxidative phosphorylation were increased (Fig. 4G). In summary, extensive alterations in gene expression modulated by SRSF1 were observed across various RNAi segments, as evidenced by heat map analysis of a curated subset of 51 genes (Fig. 4H). Collectively, our findings imply that SRSF1 is pivotal in driving the expression of genes associated with tumorigenesis throughout the transcriptome to sustain a regulatory state.

### Gene Set Enrichment Analysis (GSEA) analyzed and interpreted coordinate pathway-level changes in transcriptomics experiments

**Fig. 5:**
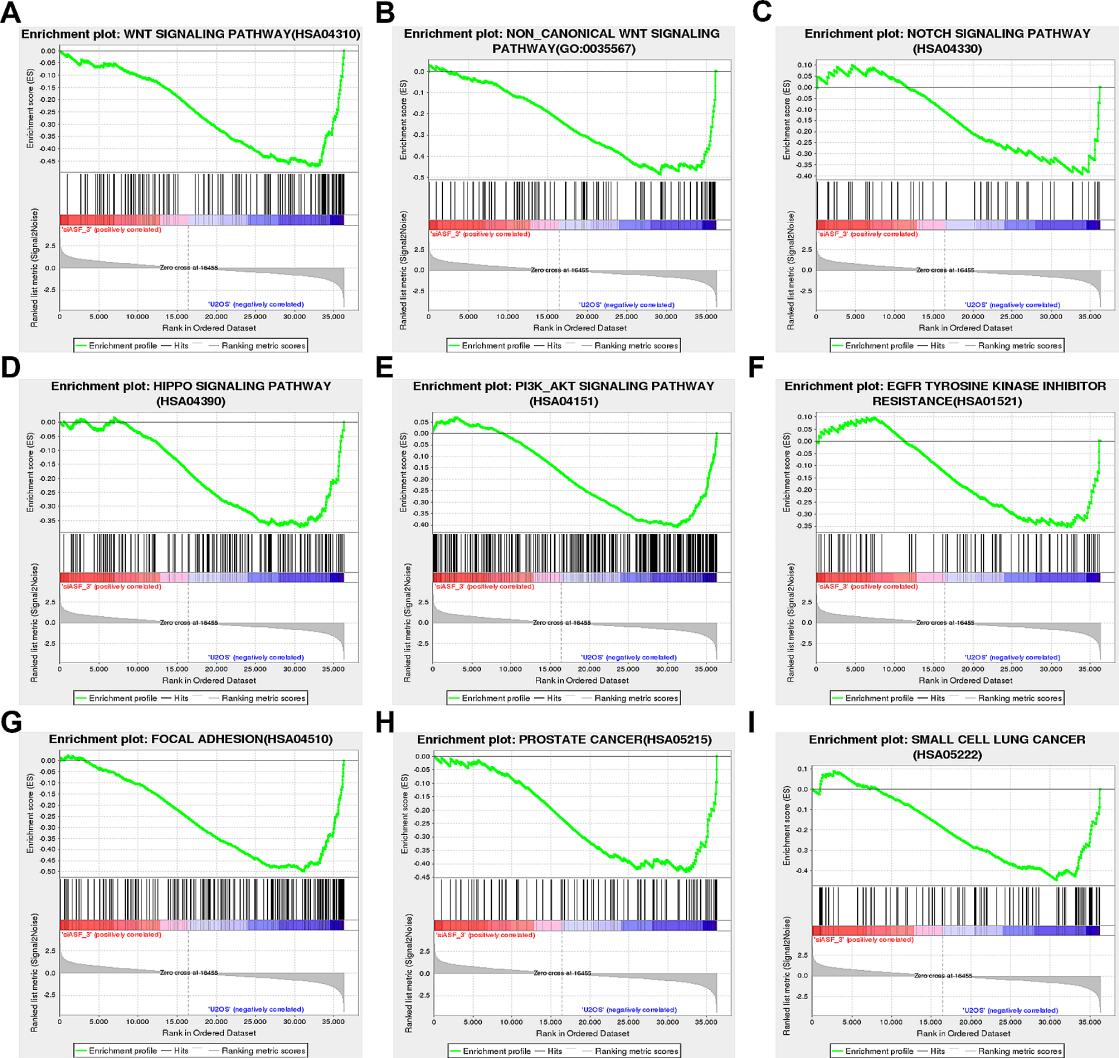
Gene Set Enrichment Analysis (GSEA) analyzed and interpreted coordinate pathway-level changes in transcriptomics experiments. GSEA employs a competitive hypothesis to test the significance of SRSF1 biological function and potential tumorigenesis in human U2OS osteosarcoma cell lines. (**A, B**) Depicts the results of the GSEA for the Wnt signaling pathway. (**C**) Depicts the results of the GSEA for the NOTCH signaling pathway. (**D**) Depicts the results of the GSEA for the HIPPO signaling pathway. (**E**) Depicts the results of the GSEA for the PI3K-AKT signaling pathway. (**F**) Depicts the results of the GSEA for the EGFR tyrosine kinase inhibitor resistance signaling. (**G**) Depicts the results of the GSEA for the Focal adhesion signaling pathway. (**H**) Depicts the results of the GSEA for the prostate cancer enrichment. (**I**) Depicts the results of the GSEA for the small cell lung cancer enrichment

By examining a range of biological conditions, genes exhibiting consistent patterns of up- or down-regulation were identified. Figure 5 illustrates the correlation between several genes and the PI3K-AKT interrelated pathways, including Wnt (Fig. 5A and B), NOTCH (Fig. 5C), HIPPO (Fig. 5D), and the PI3K-AKT pathway (Fig. 5E). We noticed that SRSF1 not only enhanced tumorigenesis and the growth of other tumors but is also implicated in drug resistance and metastasis-related signaling pathways (Fig. 5F and G). Concurrently, SRSF1 was relevant to prostate cancer and small cell lung cancer, which indicated its functions in promoting the progression of cancer (Fig. 5H and I). In summary, SRSF1 was relevant to cancer-related signaling pathways and associated with cancer progression.

### SRSF1 regulated AS events in human U2OS osteosarcoma cell lines

**Fig. 6:**
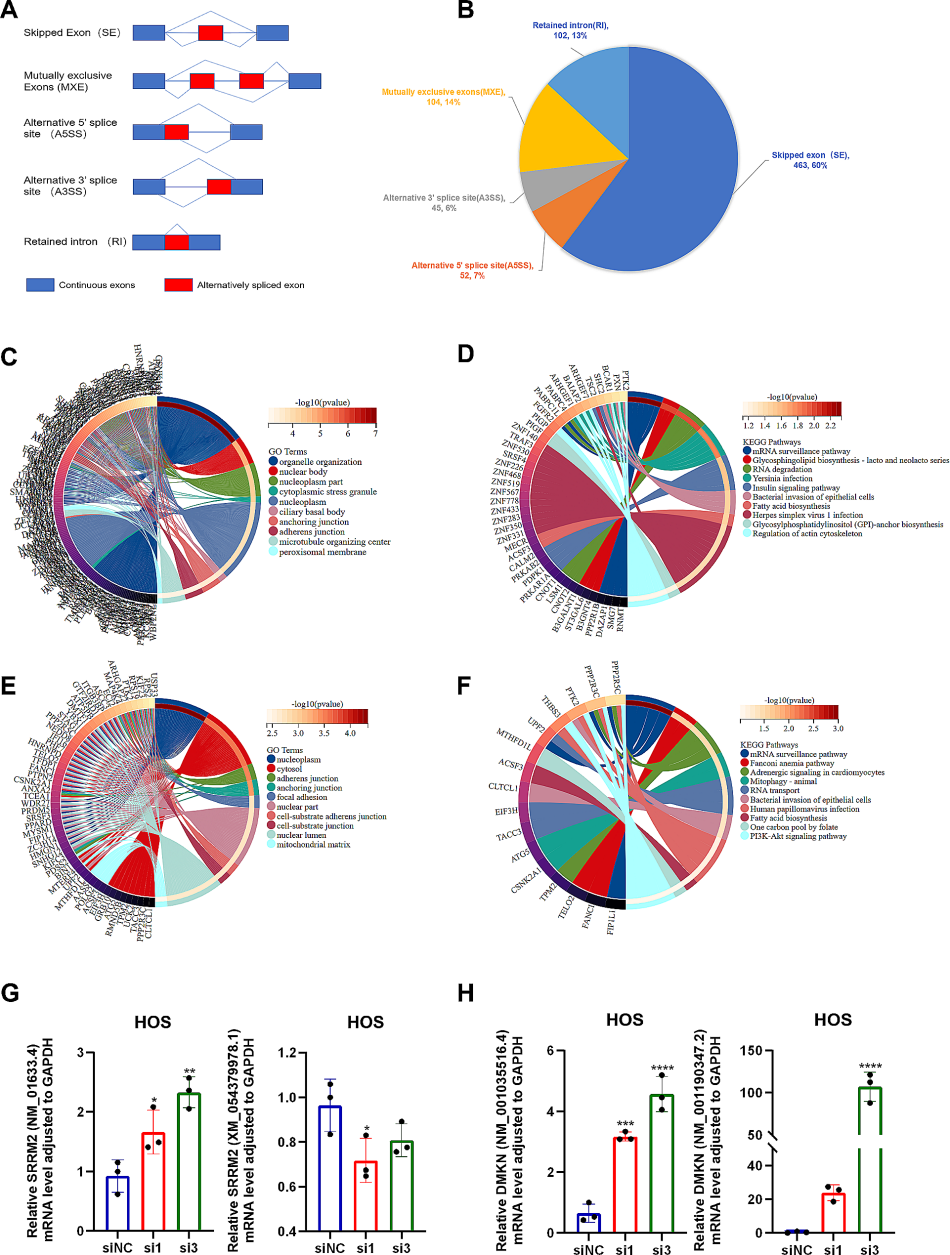
SRSF1 regulated AS events in human U2OS osteosarcoma cell lines. (**A**) The model of AS events displayed skipped Exon (SE), Mutually exclusive Exons (MXE), Alternative 5’ splice site (A5SS), Alternative 3’ splice site (A3SS), and Retained intron (RI). (**B**) Pie charts illustrated the proportion of AS events in SRSF1 RNA interference within U2OS cell lines. (**C**) The chord diagram depicted the GO functional annotations of molecules involved in SE events facilitated by SRSF1. (**D**) The chord diagram outlined the KEGG pathways of entities emerging from SE events influenced by SRSF1. (**E**) The chord diagram indicated the GO functional annotations of molecules implicated in MXE events modulated by SRSF1. (**F**) The chord diagram detailed the KEGG pathways associated with molecules derived from MXE events regulated by SRSF1. (**G-H**) qRT-PCR verifies and presents SRSF1 knockdown induced alternative splicing. Two of the predicted molecules SRRM2 and DMKN are shown. Each experiment was independently repeated three times. (*) *P* < 0.05; (**) *P* < 0.01; (***) *P* < 0.001; (****) *P* < 0.0001

The process of alternative splicing (AS) partially depends on intrinsic gene sequences and trans-acting proteins called splicing factors that recognize and bind specific target sequences in exons or introns [22]. Its regulations apply to different modes of AS (Fig. 6A). During this study, we analyzed the frequency of AS events post SRSF1 knockdown and found that Skipped Exon (SE) events were most common, constituting approximately 60% of all events, followed by Mutually exclusive Exons (MXE) with 14% of all events (Fig. 6B). The top fifty incidents of SE and MXE are respectively displayed in Supplementary Table S3-S4. The GO and KEGG analyses (Fig. 6C and D) reveal that genes exhibiting exon skipping due to SRSF1 could have associations with mRNA surveillance, RNA degradation, and maintenance of the intracellular structure. Additionally, genes undergoing mutually exclusive exons due to SRSF1 may show connections to mRNA surveillance and RNA transport (Fig. 6E and F). Specifically, the implicated genes, such as RNMT, FIP1L1, and FANCI, have been substantiated to play a role in carcinogenesis.

In further confirmation, we also observed the top three molecular alternative splicing changes, SRRM2, DMKN, and SCAT1 when downregulation of SRSF1 and found that it significantly results in alterations in two SRRM2 transcripts, two DMKN transcripts, and two SCAT1 transcripts (Fig. 6G and H, and Figure S2). Our study, therefore, discloses that SRSF1-induced AS primarily triggers SE and MXE events, thus generating a diverse range of spliceosomes tied to mRNA surveillance, RNA degradation, RNA transport, and intracellular structure.

## Discussion

In this study, we investigated the expression and functional role of SRSF1 in osteosarcoma (OS). Consistent with previous research on other types of tumors [23, 24], we found that SRSF1 expression was significantly elevated in OS tissues and cell lines. These findings suggest that SRSF1 may contribute to the development of OS.

To assess the phenotypic effects of SRSF1, we conducted a series of in vitro experiments using human OS cell lines. We observed that downregulation of SRSF1 resulted in decreased proliferation, migration, and invasion, as well as increased cell apoptosis. Conversely, overexpression of SRSF1 enhanced cell growth, migration, invasion, and anti-apoptosis. These findings indicate that SRSF1 plays a crucial role in promoting the progression of OS. In addition to SRSF1, other splicing factors have been investigated in relation to OS development. For instance, SFPQ has been shown to regulate alternative splicing (AS) of cell cycle-related genes, promoting OS progression [25]. SRSF3, another member of the SRSF family, has been found to enhance cell viability, migration, and invasion in OS cell lines [26]. On the other hand, RBM10 has been identified as a tumor suppressor in various types of tumors, including OS, inhibiting proliferation and promoting apoptosis [27]. As for SRSF1, it has been primarily studied for promoting tumor progression via alternatively splicing RNA as a splicing factor [12, 23] or for its role in binding to ncRNA to regulate the transcription of other molecules. In the present study, our findings demonstrate that SRSF1 exerts its oncogenic effects in OS through loss- and gain-of-function experiments, promoting proliferation, migration, invasion, and anti-apoptosis. These results align with previous research on the biological functions of SRSF1 in lung and breast cancer. It has been shown that SRSF1 is associated with developmental disorders in lung cancer [28], and influences patients’ radioresistance [29]. SRSF1 also exerts oncogenic roles in breast cancer partially by regulating apoptosis and cell proliferation [11, 30], and is correlated with tumor grade and poor prognosis [12]. In this study, we reported, for the first time, that SRSF1 functions as a potential oncogene in OS development. To further understand the mechanism of its carcinogenesis, we carried through transcriptome sequencing (RNA-seq).

RNA-seq is commonly employed to evaluate the differential expression of molecules in diseases, serving as a valuable tool for identifying AS [31]. In this study, we leveraged RNA-seq and identified 1701 upregulated genes and 1317 down-regulated genes in SRSF1-knockdown U2OS cell lines. Through Gene Ontology (GO) analyses, we observed potential associations between SRSF1 and the localization and function of the cytosolic ribosome, protein targeting processes, as well as the composition of the extracellular matrix (ECM) and proteinaceous extracellular matrix. These matrices have emerged as promising avenues for cancer diagnosis and therapeutic targets [32]. Previous investigations have explored their involvement in breast cancer progression and metastasis [33], association with the poor prognosis and resistance in non-small cell lung carcinoma [34], and facilitation of invasion in gastric cancer [35]. ECM has been implicated in OS development by promoting abnormal bone and vessel activity [36, 37]. This underscores the significance of SRSF1 in the progression of OS. Based on the KEGG bubble charts, it can be inferred that SRSF1 mainly participates in the PI3K-AKT signaling, Rap1 signaling, and Wnt signaling pathways, focal adhesion, and microRNAs in the development of OS. The Gene Set Enrichment Analysis (GSEA) aligns with the KEGG findings and reveals several other biological signaling pathways, such as PI3K-AKT-related pathways, Wnt, NOTCH, HIPPO, and other metastasis-related pathways, which are closely associated with SRSF1 levels. Intriguingly, the relationship between SRSF1 and some pathways has been verified in previous studies on various tumors. For instance, in hepatocellular carcinoma (HCC), SRSF1 can enhance KLF6 alternative splicing through the phosphoinositide 3-kinase (PI3K)/Akt signaling pathway, generating three splice variants that expedite tumor progression and metastasis [38]. Additionally, SRSF1 stimulates β-catenin accumulation by recruiting β-catenin mRNA and facilitating its translation in an mTOR-dependent manner, contributing to tumorigenesis [39]. Furthermore, SRSF1 activates alternative splicing of Numb, an inhibitor of the NOTCH signaling pathway, thereby augmenting NOTCH signaling and promoting tumor growth and progression [40]. Moreover, some pathways identified in our study have been validated as contributors to the malignant behavior of OS. For instance, the PI3K-AKT signaling pathway, induced by various genes, has been demonstrated to augment chemoresistance in OS [41] and expedite the progression of OS through mechanisms such as apoptosis suppression and promotion of cell proliferation, migration, and invasion [42]. The Wnt signaling pathway is also recognized for fostering OS malignancy [43], fibroblastic traits [44], distant metastases, and poor survival rates [45]. The HIPPO signaling pathway is also implicated in the initiation and advancement of OS by stimulating cell proliferation, migration, and invasion in osteosarcoma [46, 47]. Taken together, these findings suggest SRSF1 may contribute to the progression of OS via biological signaling pathways. Nonetheless, further investigations are required to elucidate the specific pathways through which SRSF1 exerts its effects.

In the context of SRSF1’s AS profile, we observed 766 AS events, with skipped exon (SE) events being dominant, constituting 60% of all events, consistent with previous research indicating SE events as the most prevalent during alternative splicing [48]. Mutually Exclusive Exons (MXE) events followed, comprising 14% of all events. Upon analysis via GO and KEGG, we inferred that SE-mediated genes through SRSF1 might be implicated in preserving mRNA stability, facilitating RNA degradation, with MXE molecules also seemingly engaged in mRNA stability and RNA transport. Past studies suggest that SRSF1 could activate MAPK signaling, partially due to the upregulation of interleukin 1 receptor type 1 (IL1R1) through alternative-splicing-regulated mRNA stability to trigger pancreatic ductal adenocarcinoma (PDAC) [23]. SRSF1 has been observed to promote nonsense-mediated mRNA decay (NMD) by recruiting UPF1, suggesting its regulatory role in gene expression and genetic diseases [49]. Additionally, assessments of Supplementary Tables 3 and Supplementary Table 4 reveal an array of molecules undergoing SE and MXE, some of which have been studied in the context of carcinogenesis in other tumor types. For example, RNMT (RNA guanine-7 methyltransferase) demonstrated an association with CDK1-cyclin B1 to sync mRNA G1 phase transcription and impact mRNA surveillance [50] and it has also been found to potentially correlate with immune cell infiltration in breast cancer (BC) [51]. FIP1L1, an MXE molecule managed by SRSF1, was reported as crucial in governing the glycolipid metabolism of GBM cells [52]. The skipping FIP1L1 (exon 13) modulated by CDYL2a was found to promote cell proliferation in breast cancer [53]. And FANCI, a DNA repair protein, was found to bind with an apoptotic effector, thereby regulating DNA repair and apoptosis [54]. PRP3 knockdown causes skipped exon 9 of FANCI and switches the FANCI splicing isoform from FANCI-12 to FANCI‐13 then resulting in delayed DNA damage repair and cell cycle G2/M arrest [55]. In this study, we confirmed that SRSF1 downregulation alters the abundance of transcripts of SRRM2, DMKN, and SCAT1, indicating a potential influence on their alternative splicing. In previous studies, changes of SRRM2 variants were reported to be closely related with Parkinson’s disease [56, 57]. Although the role of SRRM2 alternative splicing has yet been uncovered in cancers, its aberrant expression and germline mutation was found to contribute to thyroid cancer progression and recurrence [58, 59]. The DMKN gene, which harbors five splice variants (α, β, γ, δ, and ε), has been previously studied to suggest that suppressing DMKN-β/γ reduces the invasiveness and migratory capabilities of pancreatic cancer cells, potentially impacting the epithelial-mesenchymal transformation (EMT). Furthermore, DMKN-α, encoded by the variant 1 transcript (NM_001035516.4), has been identified as a potential pancreatic cancer oncogene [60, 61]. Variant 1 transcript of DMKN changes were also detected in SRSF1 knockdown cells. Besides, although alternative splicing of SCAT1 has yet been reported, its expression type has been found to be associated with cancer prognosis and immunotherapy response [62, 63]. These findings emphasize the importance of these targeted genes and/or their splice variants in tumorigenesis. Altogether, these observations propose the potential SRSF1-driven mechanism in controlling mRNA surveillance, degradation, and subsequent activation of downstream pathways to foster tumorigenesis in OS. Nevertheless, the specific biological roles of AS and its osteosarcoma-related isoforms remain to be defined. Collectively, our findings shine a light on the importance of AS events catalyzed by SRSF1 in safeguarding mRNA stability, directing degradation, and assuming essential functions in RNA transport in OS.

Despite the insightful findings, this study is not without limitations. First, an exploration into the clinical significance of SRSF1 was not conducted. Second, in vivo experiments, essential for validating the in vitro functions of SRSF1, were not executed. Finally, data obtained from transcriptome sequencing, encompassing the function of molecules and biological pathways, necessitates further validation.

## Conclusion

This investigation reveals a high expression of SRSF1 in both OS tissues and cell lines. Mechanistically, SRSF1 appears integral in the dysregulation of protein targeting processes, and the ECM, including the proteinaceous extracellular matrix, working collaterally with the PI3K-AKT, Wnt, and HIPPO pathways. Notably, AS events mediated by SRSF1, particularly SE and MXE events, and molecules such as RNMT, FIP1L1, and FANCI were identified, suggesting potential involvement in mRNA surveillance, RNA degradation, and RNA transport in the pathogenesis of OS. Collectively, our study highlights SRSF1 as a major influencer in OS progression, thereby presenting itself as a promising therapeutic target for the disease.

### Electronic supplementary material

Below is the link to the electronic supplementary material.

Supplementary Material 1

Supplementary Material 2

Supplementary Material 3

Supplementary Material 4

Supplementary Material 5

Supplementary Material 6

## Data Availability

The datasets used and/or analysed during the current study available from the corresponding author on reasonable request. The datasets generated and/or analysed during the current study are available in the [Gene Expression Omnibus (GEO)] repository, [GSE243561].
