# Supplementary material for: High expression of SRSF1 facilitates osteosarcoma progression and unveils its potential mechanisms
Source: BMC Cancer. 2024 May 12;24:580. doi: 10.1186/s12885-024-12346-y (PMC11088775; doi:10.1186/s12885-024-12346-y)
Supplement: Supplementary file 1 — Supplementary Material 1 [file 12885_2024_12346_MOESM1_ESM.docx]

**Supplementary Table 1 Top 100 upregulated genes**

| **Gene name** | **P value** |
| --- | --- |
| AC207130.1 | 0.00000000 |
| KCNIP4 | 0.00000000 |
| ANGPT2 | 0.00000000 |
| WDR66 | 0.00000000 |
| LINC01310 | 0.00000000 |
| FBXW10 | 0.00000000 |
| FAAH | 0.00000000 |
| AP001033.2 | 0.00000000 |
| TNFRSF14 | 0.00000000 |
| NEU4 | 0.00000000 |
| CDRT1 | 0.00000000 |
| AC010457.1 | 0.00000000 |
| ANKK1 | 0.00000000 |
| GOLT1A | 0.00000000 |
| PRSS8 | 0.00000000 |
| NFE4 | 0.00000000 |
| BX640514.1 | 0.00000000 |
| DIRC3 | 0.00000000 |
| ZNF165 | 0.00000000 |
| MAL2 | 0.00000000 |
| FGF21 | 0.00000000 |
| AL033527.5 | 0.00000000 |
| SCX | 0.00000000 |
| SNAPC1 | 0.00000000 |
| FAM19A5 | 0.00000000 |
| HTRA1 | 0.00000000 |
| KLF15 | 0.00000000 |
| LBX2-AS1 | 0.00000000 |
| TTC9B | 0.00000000 |
| FAM86FP | 0.00000000 |
| AL512306.2 | 0.00000000 |
| PICSAR | 0.00000000 |
| CCRL2 | 0.00000000 |
| ALOXE3 | 0.00000000 |
| HSPBAP1 | 0.00000000 |
| AC015912.3 | 0.00000001 |
| DDX39BP2 | 0.00000001 |
| AGXT | 0.00000001 |
| SPHK1 | 0.00000001 |
| KLKP1 | 0.00000001 |
| PAX8 | 0.00000001 |
| CDC42EP1 | 0.00000001 |
| AL022322.1 | 0.00000001 |
| PPP1R1C | 0.00000001 |
| FTCD | 0.00000001 |
| CSTA | 0.00000002 |
| ZNF554 | 0.00000003 |
| ACTN2 | 0.00000004 |
| NTNG2 | 0.00000004 |
| PTPN7 | 0.00000005 |
| DAGLB | 0.00000006 |
| HSD17B14 | 0.00000007 |
| C4orf19 | 0.00000008 |
| ZP3 | 0.00000008 |
| XAGE5 | 0.00000008 |
| NMRK2 | 0.00000008 |
| THOC6 | 0.00000011 |
| DTX3 | 0.00000011 |
| PKIB | 0.00000012 |
| SUN3 | 0.00000012 |
| CD22 | 0.00000012 |
| OSGIN1 | 0.00000012 |
| FOSL1 | 0.00000013 |
| CYP4V2 | 0.00000014 |
| NGF | 0.00000016 |
| VAV1 | 0.00000019 |
| PBDC1 | 0.00000020 |
| LRMDA | 0.00000020 |
| IGFN1 | 0.00000023 |
| AL392172.1 | 0.00000024 |
| AL365181.2 | 0.00000026 |
| LINC01666 | 0.00000026 |
| TRPC4 | 0.00000026 |
| BRI3 | 0.00000027 |
| GPR137B | 0.00000027 |
| BIRC7 | 0.00000031 |
| ZFPM2-AS1 | 0.00000032 |
| BEX2 | 0.00000040 |
| AC018816.1 | 0.00000047 |
| RPLP0P2 | 0.00000049 |
| ARHGAP27 | 0.00000053 |
| FLCN | 0.00000054 |
| SPNS2 | 0.00000055 |
| LINC00973 | 0.00000056 |
| BORCS7 | 0.00000060 |
| CEBPB-AS1 | 0.00000064 |
| SLC43A3 | 0.00000066 |
| RPSAP19 | 0.00000068 |
| AC026740.1 | 0.00000069 |
| C19orf54 | 0.00000069 |
| MSRB2 | 0.00000070 |
| GNPDA1 | 0.00000071 |
| ANKFN1 | 0.00000072 |
| FAM27B | 0.00000076 |
| RP9 | 0.00000077 |
| CTU1 | 0.00000078 |
| AP002761.3 | 0.00000079 |
| ARHGAP27P1-BPTFP1-KPNA2P3 | 0.00000079 |
| AL356512.1 | 0.00000082 |
| SAT2 | 0.00000085 |
