# Supplementary material for: High expression of SRSF1 facilitates osteosarcoma progression and unveils its potential mechanisms
Source: BMC Cancer. 2024 May 12;24:580. doi: 10.1186/s12885-024-12346-y (PMC11088775; doi:10.1186/s12885-024-12346-y)
Supplement: Supplementary file 2 — Supplementary Material 2 [file 12885_2024_12346_MOESM2_ESM.docx]

**Supplementary Table 2** **Top 100 downregulated genes**

| **Gene name** | **P value** |
| --- | --- |
| CA12 | 0.00000000 |
| ESM1 | 0.00000000 |
| PLAT | 0.00000000 |
| CCND1 | 0.00000000 |
| NKILA | 0.00000000 |
| PLAU | 0.00000000 |
| NEFL | 0.00000000 |
| TGFBI | 0.00000000 |
| TNFSF15 | 0.00000000 |
| LBH | 0.00000000 |
| PTPRU | 0.00000000 |
| ANXA6 | 0.00000000 |
| ITGA2 | 0.00000000 |
| PCYOX1 | 0.00000000 |
| HR | 0.00000000 |
| EMP1 | 0.00000000 |
| PGM2L1 | 0.00000000 |
| EPDR1 | 0.00000000 |
| FAM102B | 0.00000000 |
| ENPP1 | 0.00000000 |
| CCL2 | 0.00000000 |
| TMEM255A | 0.00000000 |
| IGFBP5 | 0.00000000 |
| RASSF3 | 0.00000000 |
| PODXL | 0.00000000 |
| INAVA | 0.00000000 |
| FAM20C | 0.00000000 |
| SH3TC2 | 0.00000000 |
| SMAD6 | 0.00000000 |
| ATP2B4 | 0.00000000 |
| ADAM19 | 0.00000000 |
| PDGFB | 0.00000000 |
| TRIB2 | 0.00000000 |
| DIXDC1 | 0.00000000 |
| KIAA1549L | 0.00000000 |
| PAQR4 | 0.00000000 |
| ZNF618 | 0.00000000 |
| RASSF2 | 0.00000000 |
| COL11A1 | 0.00000000 |
| LGR4 | 0.00000000 |
| PHLDA1 | 0.00000000 |
| PRICKLE2 | 0.00000000 |
| NREP | 0.00000000 |
| SMOC1 | 0.00000000 |
| CHST6 | 0.00000000 |
| C3 | 0.00000000 |
| MAML2 | 0.00000000 |
| AHNAK | 0.00000000 |
| PCDHB5 | 0.00000000 |
| LIPA | 0.00000000 |
| LHX1 | 0.00000000 |
| LCTL | 0.00000000 |
| ITGA4 | 0.00000000 |
| TIMP3 | 0.00000000 |
| EFEMP1 | 0.00000000 |
| PLEKHA7 | 0.00000000 |
| TRPA1 | 0.00000000 |
| HAPLN1 | 0.00000000 |
| AC145098.2 | 0.00000000 |
| TENM2 | 0.00000000 |
| HERC3 | 0.00000000 |
| RIN2 | 0.00000000 |
| PCDH1 | 0.00000000 |
| MYO10 | 0.00000000 |
| SFRP1 | 0.00000000 |
| GPRC5B | 0.00000000 |
| NOTCH3 | 0.00000000 |
| ZNF792 | 0.00000000 |
| FAM84A | 0.00000000 |
| MN1 | 0.00000000 |
| MLKL | 0.00000000 |
| SNAP25 | 0.00000000 |
| UCA1 | 0.00000000 |
| AL109918.1 | 0.00000000 |
| GLI2 | 0.00000000 |
| ETV1 | 0.00000000 |
| FAM117B | 0.00000000 |
| ZNF860 | 0.00000000 |
| CDKN2B | 0.00000000 |
| CELSR2 | 0.00000000 |
| PDGFRA | 0.00000000 |
| AKAP12 | 0.00000000 |
| SETBP1 | 0.00000001 |
| CTPS1 | 0.00000001 |
| IGSF9 | 0.00000001 |
| DPYSL2 | 0.00000001 |
| GJA1 | 0.00000001 |
| FLT4 | 0.00000001 |
| FGFR3 | 0.00000001 |
| MYOSLID | 0.00000001 |
| ITGB3 | 0.00000001 |
| MAML3 | 0.00000001 |
| EXT2 | 0.00000001 |
| INSYN2B | 0.00000001 |
| SOCS3 | 0.00000001 |
| TNS4 | 0.00000001 |
| JAG1 | 0.00000001 |
| PFKFB4 | 0.00000001 |
| ANGPT1 | 0.00000001 |
| LDHA | 0.00000002 |
