# Supplementary material for: High expression of SRSF1 facilitates osteosarcoma progression and unveils its potential mechanisms
Source: BMC Cancer. 2024 May 12;24:580. doi: 10.1186/s12885-024-12346-y (PMC11088775; doi:10.1186/s12885-024-12346-y)
Supplement: Supplementary file 3 — Supplementary Material 3 [file 12885_2024_12346_MOESM3_ESM.docx]

**Supplementary Table 3** **Top fifty incidents of SE**

| **Gene symbol** | **P Value** | **FDR** |
| --- | --- | --- |
| SRRM2 | 0.00000000 | 0.00000000 |
| DMKN | 0.00000000 | 0.00000000 |
| SCAT1 | 0.00000000 | 0.00000000 |
| PACSIN2 | 0.00000000 | 0.00000000 |
| WBP2NL | 0.00000000 | 0.00000000 |
| CBY1 | 0.00000000 | 0.00000000 |
| ELFN2 | 0.00000000 | 0.00000000 |
| FOXRED2 | 0.00000000 | 0.00000000 |
| CHEK2 | 0.00000000 | 0.00000000 |
| LAIR2 | 0.00000000 | 0.00000000 |
| ZNF331 | 0.00000000 | 0.00000000 |
| ZNF888 | 0.00000000 | 0.00000000 |
| ZNF350 | 0.00000000 | 0.00000000 |
| CD33 | 0.00000000 | 0.00000000 |
| FUZ | 0.00000000 | 0.00000000 |
| HSD17B14 | 0.00000000 | 0.00000000 |
| ZNF283 | 0.00000000 | 0.00000000 |
| ARHGEF1 | 0.00000000 | 0.00000000 |
| KIRREL2 | 0.00000000 | 0.00000000 |
| FAM207A | 0.00000000 | 0.00000000 |
| URI1 | 0.00000000 | 0.00000000 |
| OCEL1 | 0.00000000 | 0.00000000 |
| UCA1 | 0.00000000 | 0.00000000 |
| ADGRE5 | 0.00000000 | 0.00000000 |
| ZNF433 | 0.00000000 | 0.00000000 |
| CIRBP | 0.00000000 | 0.00000000 |
| SNHG17 | 0.00000000 | 0.00000000 |
| SPAG4 | 0.00000000 | 0.00000000 |
| HM13 | 0.00000000 | 0.00000000 |
| ELP2 | 0.00000000 | 0.00000000 |
| RNMT | 0.00000000 | 0.00000000 |
| NDUFV2-AS1 | 0.00000000 | 0.00000000 |
| DLGAP1-AS1 | 0.00000000 | 0.00000000 |
| BAIAP2 | 0.00000000 | 0.00000000 |
| USP36 | 0.00000000 | 0.00000000 |
| AP000317.2 | 0.00000000 | 0.00000000 |
| SOX9-AS1 | 0.00000000 | 0.00000000 |
| HEATR6 | 0.00000000 | 0.00000000 |
| COPZ2 | 0.00000000 | 0.00000000 |
| DONSON | 0.00000000 | 0.00000000 |
| TVP23C | 0.00000000 | 0.00000001 |
| P2RX5 | 0.00000000 | 0.00000001 |
| TLCD2 | 0.00000000 | 0.00000004 |
| SPG7 | 0.00000000 | 0.00000004 |
| CMC2 | 0.00000000 | 0.00000004 |
| BCAR1 | 0.00000000 | 0.00000005 |
| ENKD1 | 0.00000000 | 0.00000006 |
| KIAA0895L | 0.00000000 | 0.00000007 |
| CHST6 | 0.00000000 | 0.00000007 |
| ATXN2L | 0.00000000 | 0.00000015 |
