# Supplementary material for: High expression of SRSF1 facilitates osteosarcoma progression and unveils its potential mechanisms
Source: BMC Cancer. 2024 May 12;24:580. doi: 10.1186/s12885-024-12346-y (PMC11088775; doi:10.1186/s12885-024-12346-y)
Supplement: Supplementary file 4 — Supplementary Material 4 [file 12885_2024_12346_MOESM4_ESM.docx]

**Supplementary Table 4 Top fifty incidents of MXE**

| **Gene symbol** | **P Value** | **FDR** |
| --- | --- | --- |
| FLVCR2 | 0.00000000 | 0.00000000 |
| ANXA2 | 0.00000000 | 0.00000000 |
| LRRC75A-AS1 | 0.00000000 | 0.00000000 |
| RMND5B | 0.00000000 | 0.00000000 |
| ASCC3 | 0.00000000 | 0.00000000 |
| ARHGAP22 | 0.00000000 | 0.00000000 |
| RWDD3 | 0.00000000 | 0.00000005 |
| TELO2 | 0.00000000 | 0.00000006 |
| DCBLD2 | 0.00000000 | 0.00000008 |
| UBXN2B | 0.00000000 | 0.00000030 |
| AC087632.1 | 0.00000000 | 0.00000106 |
| MYSM1 | 0.00000000 | 0.00000112 |
| AC009879.3 | 0.00000000 | 0.00000131 |
| DIP2C | 0.00000000 | 0.00000199 |
| TFDP1 | 0.00000001 | 0.00000374 |
| KIF23 | 0.00000001 | 0.00000375 |
| GMFG | 0.00000001 | 0.00000382 |
| MPC1 | 0.00000001 | 0.00000521 |
| WDR27 | 0.00000003 | 0.00001109 |
| TTC23 | 0.00000025 | 0.00008260 |
| MTHFD1L | 0.00000036 | 0.00010714 |
| DMKN | 0.00000036 | 0.00010714 |
| PTK2 | 0.00000052 | 0.00014203 |
| AASS | 0.00000053 | 0.00014203 |
| DCUN1D5 | 0.00000057 | 0.00014810 |
| HNRNPD | 0.00000081 | 0.00020188 |
| SNHG12 | 0.00000089 | 0.00021234 |
| FIP1L1 | 0.00000091 | 0.00021234 |
| ECI2 | 0.00000094 | 0.00021249 |
| B3GNTL1 | 0.00000134 | 0.00028589 |
| IFI16 | 0.00000233 | 0.00048327 |
| FAM45A | 0.00000363 | 0.00071534 |
| CLTCL1 | 0.00000364 | 0.00071534 |
| TANGO2 | 0.00000402 | 0.00077131 |
| GAS5 | 0.00000684 | 0.00124663 |
| FANCI | 0.00000832 | 0.00148204 |
| PRDM5 | 0.00001301 | 0.00226226 |
| PTPN2 | 0.00001422 | 0.00241662 |
| DUXAP10 | 0.00001527 | 0.00253756 |
| SRCIN1 | 0.00001734 | 0.00281785 |
| THBS3 | 0.00001885 | 0.00299864 |
| TACC3 | 0.00001961 | 0.00305404 |
| MTERF4 | 0.00002052 | 0.00313183 |
| UPF2 | 0.00002220 | 0.00331919 |
| RPS19 | 0.00002361 | 0.00346156 |
| BIN1 | 0.00003079 | 0.00434387 |
| STAG1 | 0.00003885 | 0.00528636 |
| POLG2 | 0.00004112 | 0.00548987 |
| NEDD8 | 0.00004625 | 0.00606743 |
| C1orf21 | 0.00004733 | 0.00610187 |
