# Supplementary material for: High expression of SRSF1 facilitates osteosarcoma progression and unveils its potential mechanisms
Source: BMC Cancer. 2024 May 12;24:580. doi: 10.1186/s12885-024-12346-y (PMC11088775; doi:10.1186/s12885-024-12346-y)
Supplement: Supplementary file 5 — Supplementary Material 5 [file 12885_2024_12346_MOESM5_ESM.doc]

**Supplementary Methods**

**1. RNA-seq details**

**1.1 Sample collection and preparation**

RNA degradation and contamination was monitored on 1% agarose gels. RNA purity was checked using the NanoPhotometer and spectrophotometer (IMPLEN, CA, USA). RNA integrity was assessed using the RNA Nano 6000 Assay Kit of the Bioanalyzer 2100 system (Agilent Technologies, CA, USA).

**1.2 Library preparation**

A total amount of 1 µg RNA per sample was used as input material for the RNA sample preparations. Sequencing libraries were generated using NEBNext UltraTM. RNA Library Prep Kit for Illumina® (NEB, USA) following manufacturer’s recommendations and index codes were added to attribute sequences to each sample. Briefly, mRNA was purified from total RNA using poly-T oligo-attached magnetic beads. Fragmentation was carried out using divalent cations under elevated temperature in NEBNext First Strand Synthesis Reaction Buffer(5X). First strand cDNA was synthesized using random hexamer primer and M-MuLV Reverse Transcriptase (RNase H-). Second strand cDNA synthesis was subsequently performed using DNA Polymerase I and RNase H. Remaining overhangs were converted into blunt ends via exonuclease/polymerase activities. After adenylation of 3’ ends of DNA fragments, NEBNext Adaptor with hairpin loop structure were ligated to prepare for hybridization. In order to select cDNA fragments of preferentially 250~300 bp in length, the library fragments were purified with AMPure XP system (Beckman Coulter, Beverly, USA). Then 3 µl USER Enzyme (NEB, USA) was used with size-selected, adaptor-ligated cDNA at 37°C for 15 min followed by 5 min at 95 °C before PCR. Then PCR was performed with Phusion High-Fidelity DNA polymerase, Universal PCR primers and Index (X) Primer. At last, PCR products were purified (AMPure XP system) and library quality was assessed on the Agilent Bioanalyzer 2100 system.

**1.3 Clustering and sequencing**

The clustering of the index-coded samples was performed on a cBot Cluster Generation System using TruSeq PE Cluster Kit v3-cBot-HS (Illumia) according to the manufacturer’s instructions. After cluster generation, the library preparations were sequenced on an Illumina Novaseq platform and 150 bp paired-end reads were generated.

**1.4 Data Analysis**

Differential expression analysis of two conditions/groups (two biological replicates per condition) was performed using the DESeq2 R package (1.16.1).

Gene Ontology (GO) enrichment analysis of differentially expressed genes was implemented by the clusterProfiler R package, in which gene length bias was corrected. GO terms with corrected P value less than 0.05 were considered significantly enriched by differential expressed genes. KEGG is a database resource for understanding high-level functions and utilities of the biological system, such as the cell, the organism and the ecosystem, from molecular-level information, especially large-scale molecular datasets generated by genome sequencing and other high-through put experimental technologies (http://www.genome.jp/kegg/). We used clusterProfiler R package to test the statistical enrichment of differential expression genes in KEGG pathways.

**2. Alternative splicing analysis**

The software we use for variable shear analysis is rMATS 4.1.0 and the parameters used are the default parameters. The rMAT software can classify AS events into 5 types and can perform differential AS analysis of samples with biological replicates. Each alternative splicing event corresponds to two isoforms, namely Exon Inclusion Isoform and Exon Skipping Isoform, and the expression amount of the two Isoforms are counted and divided by their effective length to obtain the corrected expression amount, and then the Exon Inclusion is calculated the ratio of the total expression of Isoform to the two Isoforms, which is the IncLevel1 (treatment group) and IncLevel2 (control group) in the result file, and finally, the difference significance analysis was performed. Our threshold for screening differentially significant alternative splicing events is an FDR of less than 0.05.

**Supplementary Figures**

**
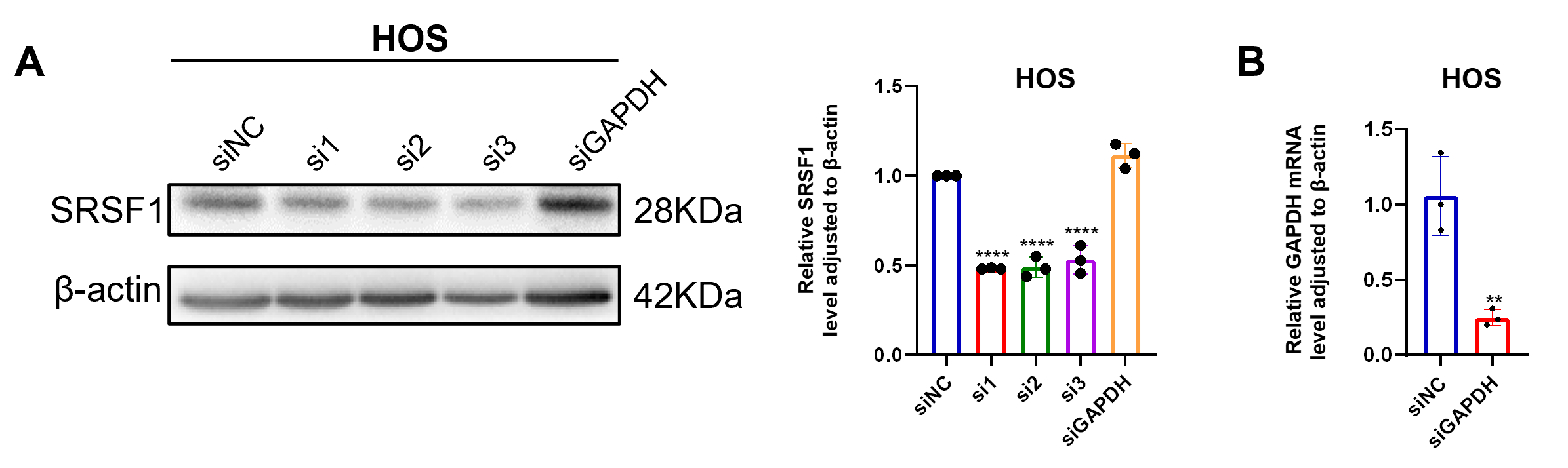
**

**Figure S1** (**A**) Western blot measured the efficiency of SRSF1 knockdown in HOS cells. (**B**) qRT-PCR measured the siRNA transfection efficiency in positive control. (*) P < 0.05; (**) P < 0.01;(***) P<0.001; (****) P<0.0001.

**
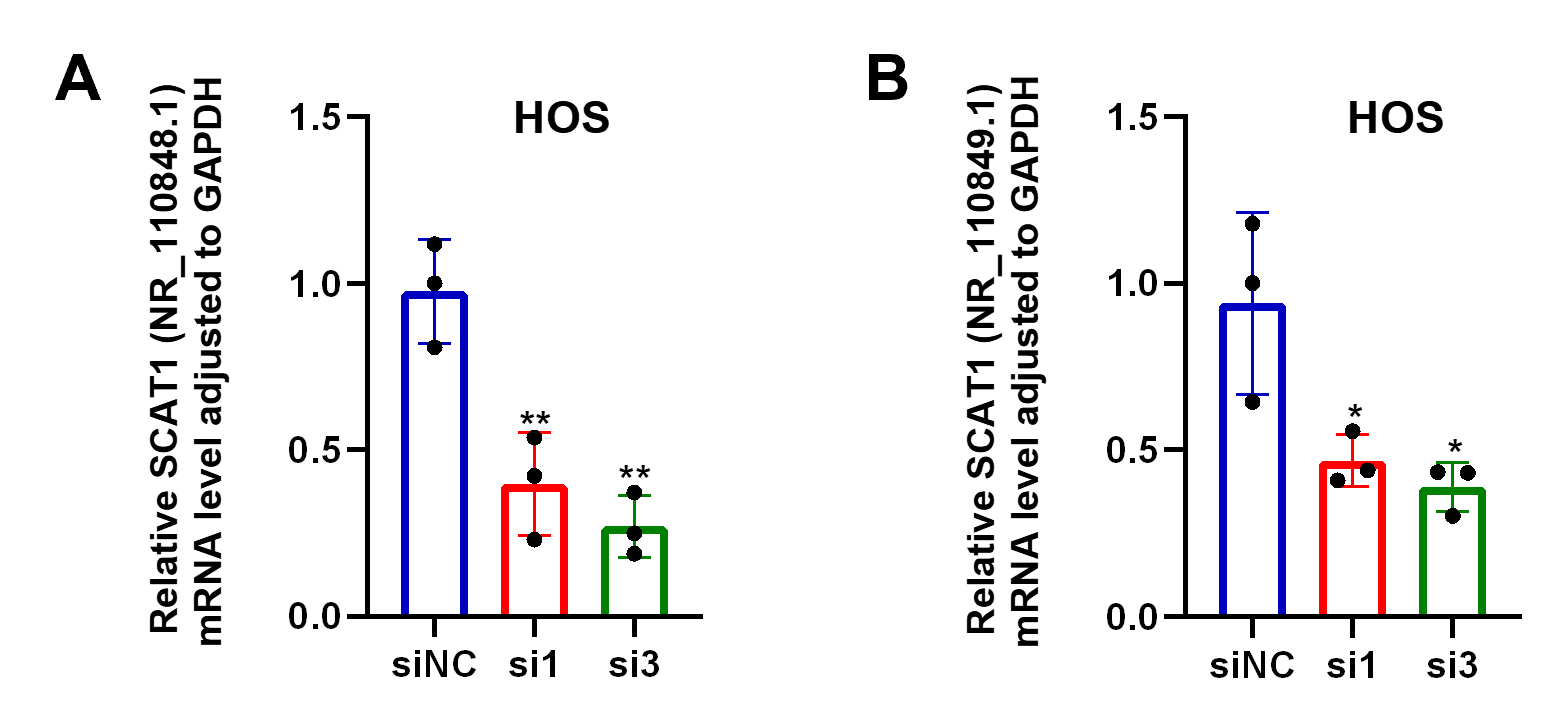
**

**Figure S2 (A, B)** qRT-PCR verifies and presents SRSF1 knockdown induced alternative splicing. Changes of two transcripts of SCAT1 are shown.
