# Supplementary material for: High expression of SRSF1 facilitates osteosarcoma progression and unveils its potential mechanisms
Source: BMC Cancer. 2024 May 12;24:580. doi: 10.1186/s12885-024-12346-y (PMC11088775; doi:10.1186/s12885-024-12346-y)
Supplement: Supplementary file 6 — Supplementary Material 6 [file 12885_2024_12346_MOESM6_ESM.docx]

**WB results:**


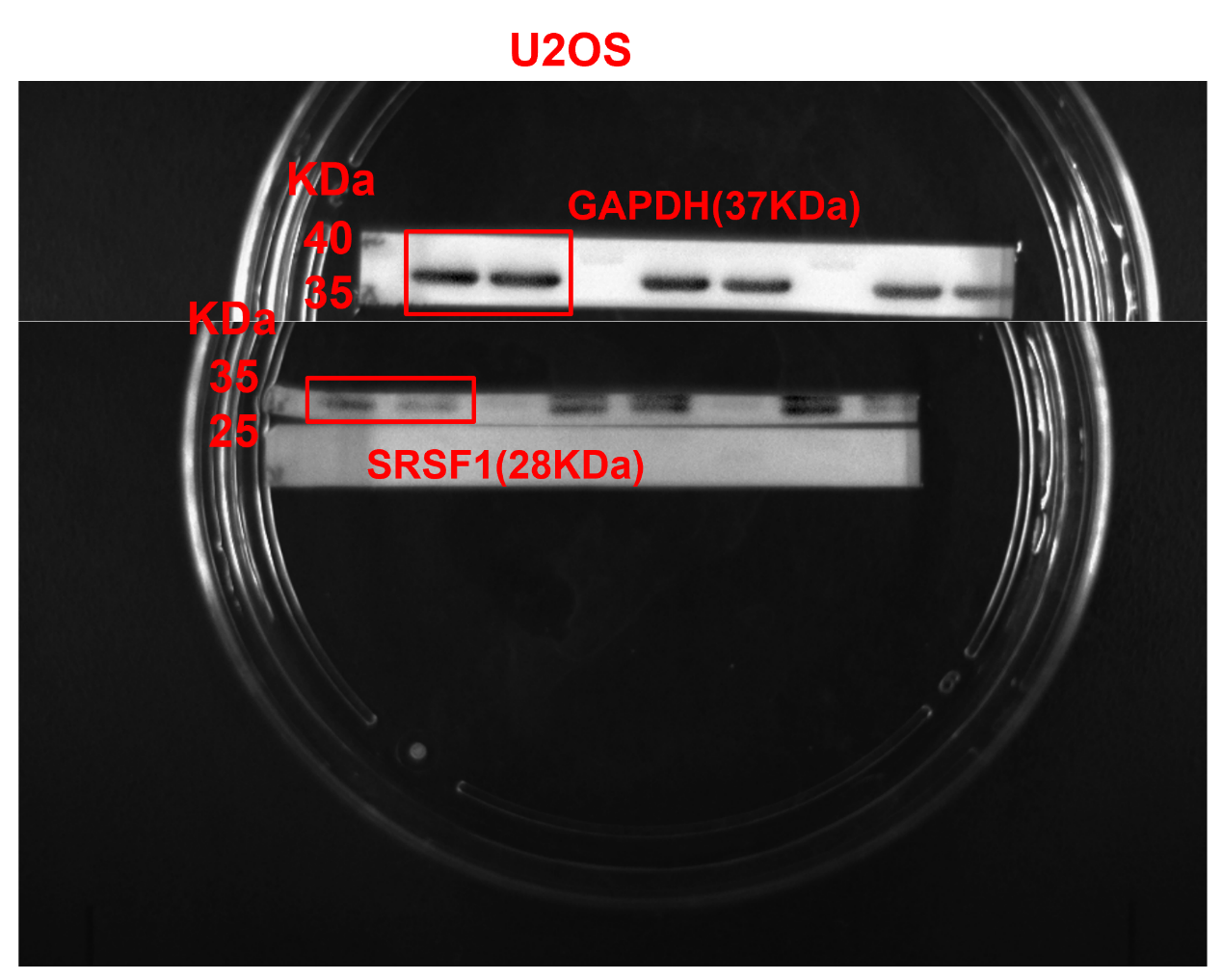


**Figure S3 This is the original exposure image of Figure 2B.** The upper panel is the GAPDH and the lower panel is the SRSF1, which are from the same gel. Displays the full-length gels of GAPDH protein and SRSF1 protein bands in SRSF1 and Vector. We used the protein marker from ThermoFisher (cat:26616). We used the ECL chemiluminescence imaging method and exposure at the same time and under the same environment, but the development strength was slightly different. Cropped 2 grouping blots are displayed in Figure 2B, which shows in order of ‘SRSF1, Vector’.


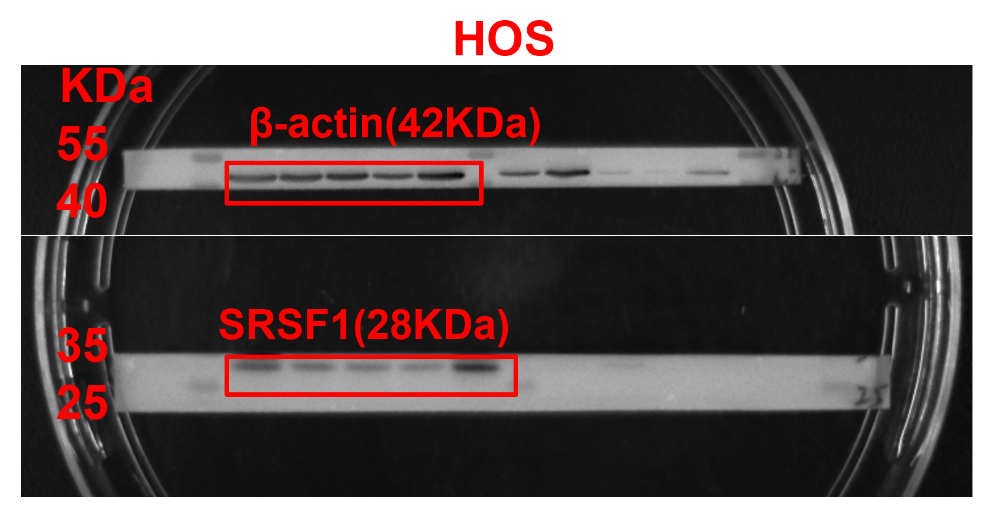


**Figure S4 This is the original exposure image of Figure 3A and Figure S1A.** The upper panel is the β-actin and the lower panel is the SRSF1, which are from the same gel. Displays the full-length gels of β-actin and SRSF1 protein bands in siNC, si1, si2, si3, and siGAPDH. We used the protein marker from ThermoFisher (cat:26616). We used the ECL chemiluminescence imaging method and exposure at the same time and under the same environment, but the development strength was slightly different. Cropped 4 grouping blots are displayed in Figure 3A, which shows in order of ‘siNC, si1, si2, and si3’. Cropped 5 grouping blots are displayed in Figure S1A, which shows in order of ‘siNC, si1, si2, si3, and siGAPDH’.

~~
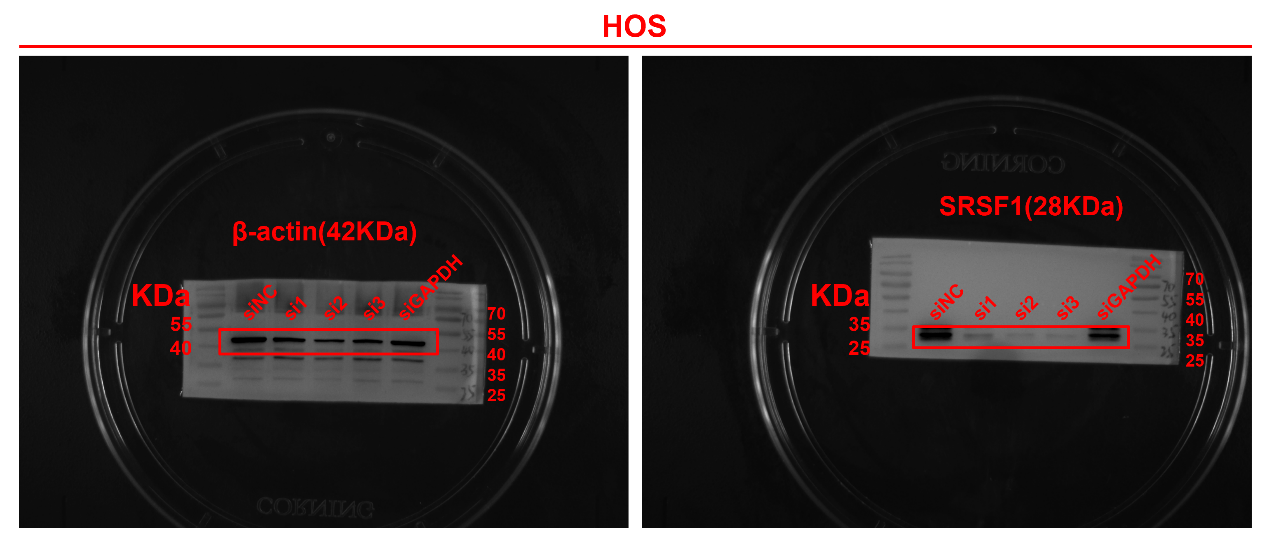
~~

**Fig S5. This is one fuller-length, original, unprocessed blot performed with samples in order of ‘siNC, si1, si2, si3, and siGAPDH’ for β-actin (left panel) and SRSF1 (right panel) in HOS cell lines.** We showed the β-actin which was the internal reference gene in order of ‘siNC, si1, si2, si3, and siGAPDH’ in left panel and the SRSF1 RNAi effect in order of ‘siNC, si1, si2, si3, and siGAPDH’ in right panel when we used western blot to measure the efficiency of SRSF1 knockdown in HOS cells.
